# Pan African Medical Journal (PAMJ) – African Field Epidemiology Network (AFENET): A partnership for the future of medical publishing in Africa

**Published:** 2008-11-11

**Authors:** Raoul Kamadjeu, David Mukanga, Landry Tsague

**Affiliations:** 1 Managing editors of the Pan African Medical Journal,; 2 Executive director of the African Field Epidemiology Network.

**Keywords:** African medical journals, open access

In October 2008, the executive director of the African Field Epidemiology Network (AFENET), David Mukanga and the managing editor of the Pan African Medical Journal (Pamj), Raoul Kamadjeu, signed a memorandum of understanding establishing the terms for future involvement of AFENET in open access publishing through the Pamj.

According to the agreement, AFENET will become the institutional backbone of the journal and will host an editorial office of the journal in its headquarters in Kampala, Uganda. Both parties will work toward achieving and maintaining the highest quality of published products with the aim of positioning the journal as one of the leading biomedical publication on the African continent.

Established in 2005 as a non-profit organization, AFENET is working in partnership with Ministries of Health, non-government organizations, international agencies, private sector, and other public health agencies to enhance or develop African nations’ applied epidemiology capacity. The mission of AFENET is to improve the health of people in Africa by strengthening and expanding applied epidemiology and laboratory capacity on the African continent. From its humble beginnings in 2005, AFENET has grown from a four-country organization to a 9-member country organization with operations in more than 15 African countries. AFENET’s achievements include: technical and financial support to existing and new Field Epidemiology Training programs; support for capacity building through short courses in French West Africa (Togo, Mali, Niger and Burkina Faso), Nigeria, Tanzania, Ghana, Zimbabwe, Uganda; Participation of Field Epidemiology Training Program (FETP) trainees in outbreak and other field investigations including Cholera in Kenya, Ebola, Marburg, plague, in Uganda, H5N1 in Nigeria; support to Ministries of Health in public health surveillance, and outbreak investigation; supporting countries to build capacity for implementation of the International Health Regulations, and response to the threat of Zoonotic diseases.

Planned activities for 2009 include: support for FETP trainees to undertake operations research through a competitive grant making process (AFENET Trainee grants program); establishment of collaborating centers for Non Communicable Disease Surveillance; a center of excellence for management and leadership training in Accra, Ghana; and, Experience@afenet, a fellowship program that provides an opportunity for graduates of African FETPs and other public health professionals from around the world to work with AFENET in areas of global health.

In addition to positioning Pamj as one of the premier open access biomedical journal on the African continent, this convergence of missions will leverage the potential within AFENET to stimulate and expand open access biomedical publishing from African scientist in the years to come. We are more than ever before aware of the urgent need to bring to higher standards, biomedical publishing in Africa. AFENET training expertise in scientific writing and alumni network will support this endeavor through mentoring junior African writers. One of our mid-term objectives is indexation of Pamj contents to major biomedical indexes, including the US National Library of Medicine by the end of the year 2009.

Finally, we welcome partnerships with other existing medical journals on the African continent as we move into the implementation stage of the partnership between Pamj and AFENET.

